# Importance of experience in transoesophageal echocardiographic evaluation of vegetation size in patients with infective endocarditis: a reliability study

**DOI:** 10.1093/ehjimp/qyae024

**Published:** 2024-04-09

**Authors:** Louise Schmidt, Lauge Østergaard, Frederik Fasth Grund, Line Schmidt, Jesper James Linde, Lars Køber, Emil L Fosbøl

**Affiliations:** The Heart Centre, Department of Cardiology, Copenhagen University Hospital, Rigshospitalet, Blegdamsvej 9, 2100 Copenhagen, Denmark; The Heart Centre, Department of Cardiology, Copenhagen University Hospital, Rigshospitalet, Blegdamsvej 9, 2100 Copenhagen, Denmark; The Heart Centre, Department of Cardiology, Copenhagen University Hospital, Rigshospitalet, Blegdamsvej 9, 2100 Copenhagen, Denmark; Department of Emergency Medicine, Bispebjerg and Frederiksberg Hospital, Nielsine Nielsens Vej 41e, 2400 Copenhagen, Denmark; The Heart Centre, Department of Cardiology, Copenhagen University Hospital, Rigshospitalet, Blegdamsvej 9, 2100 Copenhagen, Denmark; The Heart Centre, Department of Cardiology, Copenhagen University Hospital, Rigshospitalet, Blegdamsvej 9, 2100 Copenhagen, Denmark; The Heart Centre, Department of Cardiology, Copenhagen University Hospital, Rigshospitalet, Blegdamsvej 9, 2100 Copenhagen, Denmark; Department of Clinical Medicine, University of Copenhagen, Blegdamsvej 3B, 2200 Copenhagen, Denmark

**Keywords:** interrater, reliability, infective endocarditis, vegetation size, transoesophageal echocardiography, agreement

## Abstract

**Aims:**

Vegetation size assessed by transoesophageal echocardiography (TOE) is a decisive metric in guiding surgical intervention and prognosis in patients with definite infective endocarditis (IE). The aim of this study was to assess the impact of echocardiographic experience on the reliability and reproducibility of TOE measurements of vegetations in patients with IE.

**Methods and results:**

Twenty-nine raters from a cardiac department at a tertiary centre were divided into three groups according to echocardiographic experience: experts, cardiologists, and novices. All raters were instructed to measure the maximum length of vegetations in 20 different TOE exams. Interrater agreement was evaluated using intraclass correlation coefficient (ICC), one-way analysis of variance, Kruskal–Wallis test, and Bland–Altmann plots. Reliability was assessed by minimal detectable change (MDC). All measurements were compared with the measured size agreed on by the multi-disciplinary IE team.

There was an overall significant interrater variance between the three groups (*P* < 0.001). The variance was 10.1, 14.8, and 21.7 for the experts, cardiologists, and novices, respectively. ICC was excellent for experts (96.3%) and cardiologists (93.7%) and good for novices (84.6%). The three groups tended to measure smaller than the endocarditis team. MDC was 2.6 mm for experts, 3.3 mm for cardiologists, and 3.6 mm for novices.

**Conclusion:**

The study showed good to excellent intraclass correlation but high dispersion in all groups. Variance decreased with higher experience. Our findings support current recommendations that complicated cases should be cared for by the multi-disciplinary endocarditis team and underline the importance of echocardiographic expertise when evaluating and measuring vegetations in patients with IE.

## Graphical Abstract

**Graphical abstract:**
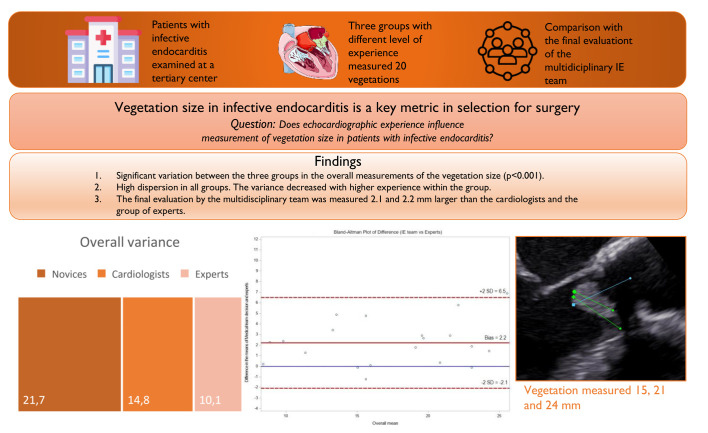

## Introduction

Infective endocarditis (IE) is a serious condition associated with severe morbidity and mortality.^1^ Diagnosing IE can be difficult and echocardiography is an essential tool in the diagnostic work-up and monitoring of the disease. One major criterion according to the ISCVID-Duke criteria is a positive finding of an intracardiac involvement, often the presence of vegetations.^2,3^

The size of the vegetation has been identified as a prognostic factor in the prediction of systemic embolization in patients with IE.^3^ Numerous studies have suggested that IE patients with vegetations >10 mm have an increased risk of embolic events, valvular destruction, and death if treated with antibiotics only compared with surgery.^3–8^ Even though the evidence level is low, European and American IE guidelines use the 10 mm cut-off as a prognostic tool for treatment strategy.^9–12^ Guidelines state that surgery should be considered for patients with left-sided IE vegetations >10 mm and recommend surgery for patients with isolated vegetations >30 mm.^9–12^ Even though vegetation size is rarely the only parameter for surgery, it was considered an indication for surgical intervention in 48% of patients undergoing surgery for IE in a large observational European study.^13^ Thus, underlining the importance of this key echocardiographic metric.

Transoesophageal echocardiography (TOE) is the imaging modality of choice for evaluating vegetation size.^9,11,12^ However, TOE is highly operator-dependent especially when it comes to identifying echocardiographic outliers and differentiating bacterial vegetations from valve structures, pre-existing valvular lesions, and echocardiographic artefacts.^9^ It is important that the measurements are reliable and reproducible to ensure that guidelines are used optimally. In fact, a careful standardized measurement of vegetation size is already recommended as part of risk stratification and follow-up during antibiotic treatment.^14^

The aim of this study was to investigate the magnitude of interrater variability related to echocardiographic experience in TOE-acquired measurements of vegetation size in patients with IE.

## Methods

### Study design

This study was reported according to the Guidelines for Reporting Reliability and Agreement Studies statement.^15^ It was a prospective single-centre study on interrater agreement conducted at a highly specialized cardiac department of the Copenhagen University Hospital, Rigshospitalet. Medical doctors (MDs) with various echocardiographic experiences were instructed to analyse TOE images and to measure vegetation size in patients with IE.

### Raters

The raters of the study were MDs with different levels of echocardiographic expertise employed at the Copenhagen University Hospital, Rigshospitalet. In the echocardiographic department, there are 11 echocardiographic specialists, one of them being part of the specialized multi-disciplinary endocarditis team. In order to identify differences in interrater variability according to experience, we categorized 3 groups of each 10 raters based on levels of echocardiographic experience. The final evaluation was made by 9 experts, 9 cardiologists, and 10 novices and the echocardiographic expert as part of the final evaluation by the IE team. One expert and one cardiologist were not able to participate in the final evaluation within the time frame of the project.

The first group, referred to as the expert group, were highly experienced MDs from the echocardiographic department. Each expert had extensive experience with TOE and transthoracic echocardiography (TTE) and evaluates and performs echocardiography daily.

Each expert was assessing approximately 40 IE patients each year.

The second group, referred to as the cardiologist group, were cardiologists with other subspecialties, i.e. arrhythmia specialist, percutaneous cardiac intervention specialists, specialists in heart failure, etc. This group had different levels of expertise in performing TOE and TTE but was very experienced in assessing and analysing basic echocardiography. They had limited to moderate experience in evaluating and measuring vegetations. The third group, referred to as the novice group, were cardiology residents, i.e. not yet specialized and with limited practical hands-on experience with echocardiography. The novice group had limited to none experience with performing and limited experience in analysing echocardiography.

None had evaluated or measured vegetations in TOE before.

Also, we compared all measurements from the three groups with the final evaluation measured by the specialized multi-disciplinary endocarditis team at Rigshospitalet. This team will be referred to as the IE team. The echocardiographic specialist from this team evaluates more than 350 IE TOE exams each year.

All the raters received oral or written information about the purpose and method of the study prior to their consent for participation. On the day of participation, all raters were introduced to the study protocol. The raters did not receive any information about how to measure the vegetations but were told to measure them as they would do in daily clinical practice.

### Echocardiography

The analyses and measurements of the echocardiographic exams took place at the Cardiac Imaging Department at Copenhagen University Hospital, Rigshospitalet. The TOEs were analysed on an online workstation using Philips—IntelliSpace Cardiovascular software.

Each rater was presented to one full TOE exam at a time having a maximum of 3 min to complete the measurement of the maximum vegetation length. In cases where more than one rater completed the measurements in the same room on different computers, they were constantly observed and ensured not to influence each other. If the raters found more than one vegetation on the examination, they were instructed to measure the largest one. All measurements were noted on a piece of paper with numbers corresponding to the echocardiographic exams. The raters did not receive any clinical information about the patients or the hospitals where the examinations were performed. They did not receive any information about the location of the vegetation or how to perform the measurements.

Furthermore, the raters were asked to report subjective information on experience with TTE and TOE, experience with measuring vegetations in IE patients, and the use of echocardiography in their daily clinical practice.

### Data material

The echocardiographic images came from conveniently chosen patients diagnosed with IE admitted to Rigshospitalet from June 2021 to June 2022. The initial inclusion criteria were patients with definite IE. Definite IE was defined according to the modified Duke Criteria as a positive finding of a vegetation on TOE.^2^

We wanted to include 20 different TOE exams from patients with IE. We wanted to include a variety of lesions including native valves and prostheses as well as various locations (e.g. mitral, aortic, and tricuspid valves). We allowed moderate-quality exams to exemplify real-life circumstances.

We used the echocardiographic exams that were used to diagnose the patient with IE. Thus, the TOE was performed at various hospitals throughout the region and the quality of the images differed. In five patients, we could not obtain the initial diagnostic TOE, and therefore the first available TOE after the IE diagnosis was used.

Furthermore, basic information about the patients were recorded from the patients’ medical charts. The study follows all national ethical principles regarding register-based studies issued by the National Ethics Committee in Denmark. Informed consent is waivered, and the Danish Data Protection Agency has approved data acquisition (P-2020-92).

### Statistics

Continuous variables were expressed as median with interquartile range (IQR) and mean ± standard deviation (SD). Categorical variables were expressed as percentages. Interrater reliability and agreement were assessed using intraclass correlation coefficient (ICC) with a 95% confidence interval, Kruskal–Wallis test, one-way analysis of variance, and Bland–Altmann plots. The ICC values were calculated using a two-way mixed model with absolute agreement.^16^ ICC values <0.50 indicate poor reliability, values between 0.50 and 0.75 indicate moderate reliability, values between 0.75 and 0.90 indicate good reliability, and greater than 0.90 are indicative of excellent reliability.^16^

Supplementary analysis was performed on the original data to rearrange the measurements into categories of <10, 10–15, and >15 mm. Each measurement was placed into the corresponding category and the absolute agreement was calculated. The level of statistical significance was set to a *p*-value of <0.05.

The difference between groups and the IE team was assessed by Bland–Altmann plots. The reliability was calculated in two different ways. The minimal detectable change (MDC) was calculated as the standard error of the mean∗1.96∗√2 and the percentage change was calculated ([highest measurement − lowest measurement]/mean)*100. Statistical analyses were performed using SAS version 9.4 and IBM SPSS Statistics 25. No outliers were removed from the statistical analyses.

## Results

Echocardiograms from a total of 20 patients with definite IE vegetations on TOE were evaluated and measured by 29 raters resulting in a total of 580 measurements.

### Data material

Among the 20 patients, there were 16 males (80%). The mean age of the patients at the time of diagnosis was 56.5 years. The number of echocardiographic video loops varied between exams from 22 to 91 with a mean of 48 loops. The bacteria detected from blood samples at diagnosis were *Staphylococcus aureus* (*n* = 6), *Enterococcus faecalis* (*n* = 2), *Haemophilus parainfluenzae* (*n* = 1), *Streptococcus mitis* (*n* = 1), *Enterococcus faecium* (*n* = 1), *Streptococcus sanguinis* (*n* = 2), *Staphylococcus epidermidis* (*n* = 1), *Staphylococcus lugdunensis* (*n* = 1), and *Streptococcus bovis* (*n* = 1) and four with no identification of the pathogen (culture negative).

A total of 11 patients underwent surgery.

Out of 20 patients, there were 18 with only one vegetation. These were eight native mitral valves, two prosthetic mechanical aortic valves, three prosthetic biological aortic valves, one on a pace wire, one native tricuspid valve, and three native aortic valves. One patient had two vegetations, one native mitral valve and one native aortic valve. Finally, one patient had three vegetations on the native mitral, native aortic, and native tricuspid valves, respectively.

### Group variation

**Figure 1:**
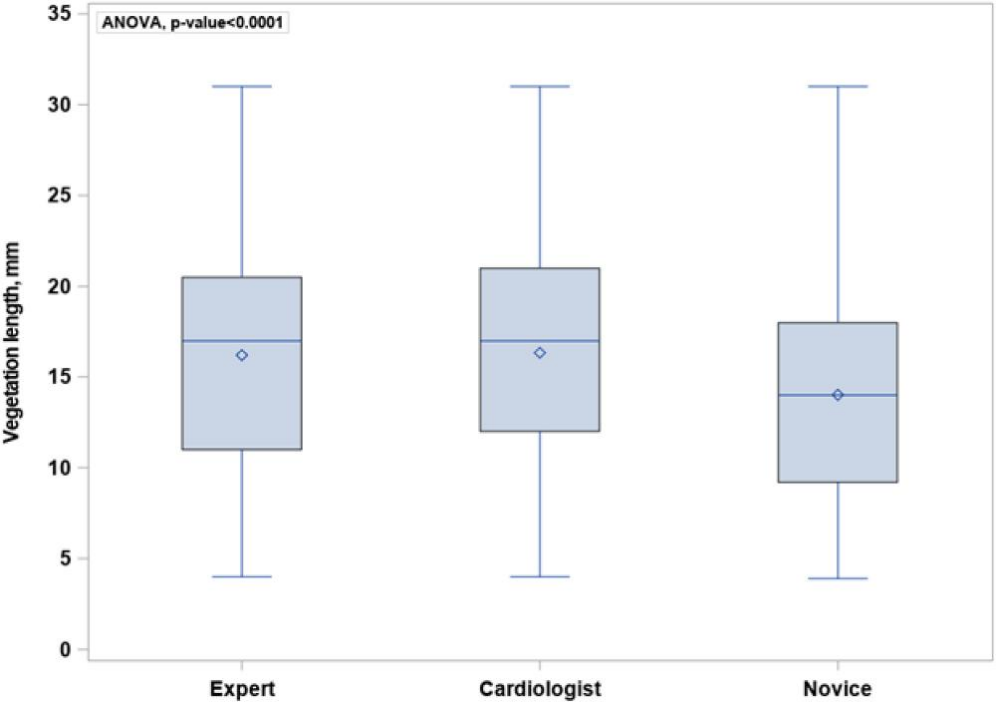
Boxplot showing significant variation between the three groups in the overall measurements of the vegetation size (*P* < 0.001). The mean length ± SD of the vegetations measured was 16.2 ± 2.8 mm for experts, 16.3 ± 3.6 mm for cardiologists, and 14.0 ± 4.1 mm for novices. The boxplot shows the mean (diamond), the median, upper and lower quartile (square), and the upper and lower extreme (±2 SD). The bullets illustrate observations outlying upper and lower fence.

*Figure 1* shows a significant variation between the three groups in the overall measurements of the vegetation size (*P* < 0.001). The mean ± SD was 16.2 ± 2.8 mm for experts, 16.3 ± 3.6 mm for cardiologists, and 14.0 ± 4.1 mm for novices. The mean vegetation length measured by all groups was 15.5 mm.

The median length (IQR) of the vegetations measured was 15.9 (3.4), 16.6 (3.8), and 13.7 (4.4) mm for experts, cardiologists, and novices, respectively. The median vegetation length measured for all groups was 15.4 mm. Supplementary data online, *Figure S1* illustrates the variation in each vegetation between the three groups.

As shown in *Table 1*, the mean variance was 10.1 for the experts, 14.8 for the cardiologists, and 21.7 for the novices. Interobserver comparison within the groups were assessed with ICC. For the experts, the ICC was 95.8% (92.4–98.1%) indicating excellent correlation. For cardiologists, the ICC was 93.7% (88.2–97.1%) indicating excellent correlation. For the novice group, the ICC were 84.6% (72.4–92.9%) indicating a good correlation. MDC was 2.6 mm for the expert group, 3.3 mm for the cardiologists, and 3.6 mm for the novice group. The percentage change was 52.3% for the experts, 64.3% for the cardiologists, and 91.5% for the novices. Supplementary analysis in which original data were rearranged into categories of <10, 10–15, and >15 mm was performed, and in this case, absolute agreement was 80% for the experts, 75% for the cardiologists, and 67% for the novices.

**Table 1:** Overview of reproducibility and reliability measurements

Group	Experts	Cardiologists	Novices
Median **±** IQR	15.9 ± 3.4 mm	16.6 ± 3.8 mm	13.7 ± 4.4 mm
Mean **±** SD	16.2 ± 2.8 mm	16.3 ± 3.6 mm	14.0 ± 4.1 mm
ICC (95% LOA)	95.8% (92.4–98.1)	93.7% (88.2–97.1)	84.6% (72.4–92.9)
Variance	10.1	14.8	21.7
MDC	2.61 mm	3.30 mm	3.56 mm

IQR, interquartile range; SD, standard deviation; ICC, intraclass correlation coefficient; LOA, limit of agreement; MDC, minimal detectable change.

**Figure 2:**
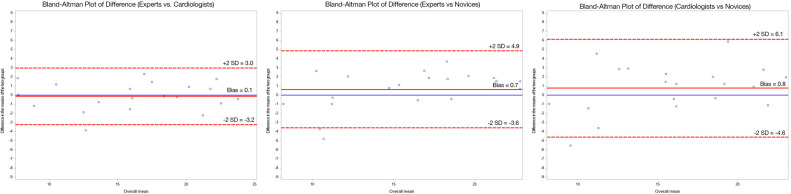
Three Bland–Altmann plots comparing the experts and the cardiologists, the experts and the novices, and the cardiologists and the novices, respectively. Blue line marks a 0 mm difference. Red full line illustrates bias where novices systematically underestimated the length compared with experts and cardiologists. Red dotted line marks ±2 SD (95% LOA). Graph A compares experts and cardiologists. Cardiologists measured a mean of 0.1 mm larger than the Experts indicating no overall measurement difference between the groups. The 95% LOA was −3.2 to 3.0. Graph B compare experts and novices. The experts measured a mean of 0.7 mm larger than the novices. The 95% LOA was −3.6 to 4.9. The last graph compared the cardiologists and the novices. The cardiologists measured 0.8 mm larger than the novices with a 95% LOA of −4.6 to 6.1. There is no systematical bias in either of the graphs.

Three Bland–Altmann plots are shown in *Figure 2* comparing the experts and the cardiologists, the experts and the novices, and the cardiologists and the novices, respectively. There was no significant difference in the mean measurement between the cardiologists and the experts, and there was no systematic bias between these two groups. The novices had a significant systematic underestimation compared with both the experts and the cardiologists.

The 95% limit of agreement (LOA) was −3.2 to 3.0 mm for experts and cardiologists, −3.6 to 4.9 mm for experts and novices, and −4.6 to 6.1 mm for cardiologists and novices.

**Figure 3:**
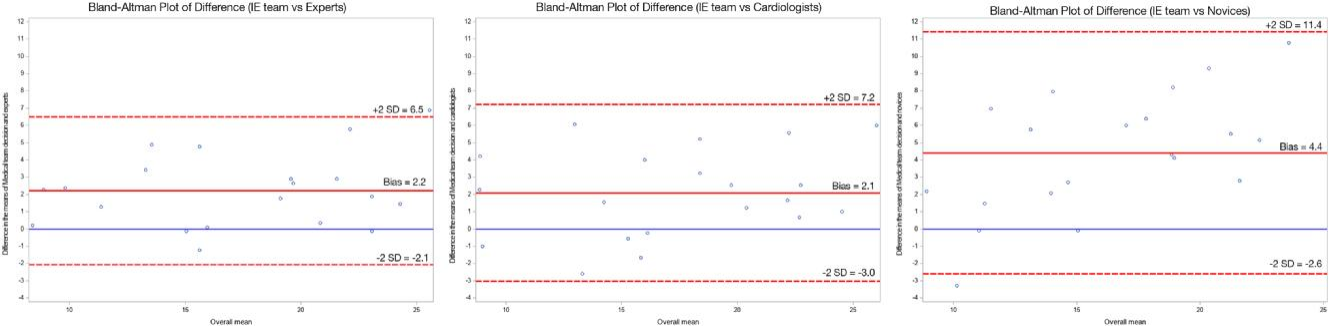
Three Bland–Altmann plots comparing each group with the multi-disciplinary IE team. Blue line marks a 0 mm difference. Red full line illustrates bias where the IE team in general measured larger than the three groups. Red dotted lines marks ±2 SD (95% LOA).

*Figure 3* shows three Bland–Altmann plots illustrating the difference between each group and the multi-disciplinary IE team. In 17 out of 20 cases, the IE team measured larger than the experts, with a mean of 2.2 mm per vegetation. The 95% LOA was −2.1 to 6.5 mm. The IE team measured 15 out of 20 vegetations larger than the cardiologists, with a mean of 2.1 mm per vegetation. The 95% LOA was −3.0 to 7.2 mm. Lastly, the IE team measured 17 out of 20 vegetations larger than the novices, with a mean of 4.4 mm per vegetation. The 95% LOA was −2.6 to 11.4 mm. The absolute difference from the IE team per vegetation were 3.7, 3.8, and 6.1 mm for the experts, cardiologists, and novices, respectively.

**Figure 4:**
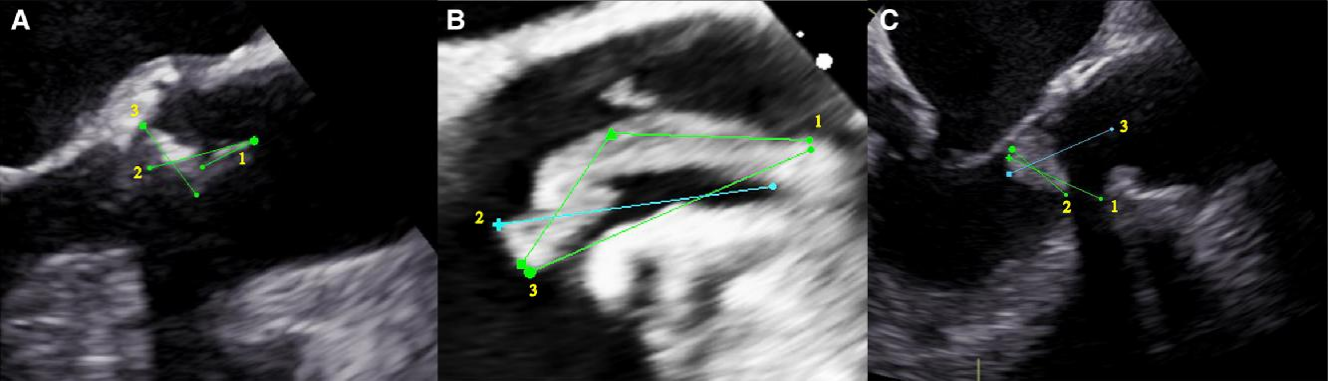
Three examples of measurements done by different raters show how vegetations can be measured with various techniques. A: Aortic native valve IE. Number one measures 8 mm, number two measures 15 mm, and number three measures 12 mm. B: Pace wire IE. Number one measures 29 mm, number two measures 22 mm, and number three measures 25 mm. C: Mechanical aortic valve IE. Number one measures 22 mm, number two measures 15 mm, and number three measures 24 mm.

*Figure 4* illustrates three examples of TOE measurements were performed by different raters. The examples were chosen to illustrate the difficulties of measuring the size of the vegetation where vegetation shape and echocardiographic shadowing are making the measurement an operator-dependent assessment.

## Discussion

We investigated how three groups of MDs with various echocardiographic experiences measured vegetation size in TOEs from 20 patients with IE. We found significant interrater variability in measuring vegetation size with decreasing variance with higher experience. The ICC was excellent within the expert and cardiologist group, and good within the novice group. Compared with the IE team, all groups tended to generally measure vegetations smaller.

Our study showed that the dispersion was decreasing with a higher level of experience indicating a better agreement and hence reproducibility. The variance increased by 45% between the experts and the cardiologists, and a further 46% between the cardiologists and novices.

A study from 2022 showed fair to good interrater agreement of 9 experienced echo readers evaluating 73 left-sided valvular findings on TOE.^17^ The overall percentage agreement was 58% for aortic valve vegetations and 68% for vegetations at the mitral valve when categorizing the vegetations into small, medium, or large. Furthermore, the study evaluated TOEs of suspected IE patients, arranging them into ‘definite’, ‘possible’, or ‘no’ IE. The agreement was lowest in cases that were interpreted as ‘possible’ IE. In our study, all patient cases were diagnosed with definite IE, and therefore raters were aware that the echocardiography included a vegetation. This may have influenced our results differentially across the groups, where the less experienced raters would tend to search for something to measure, sometimes measuring artefacts instead of vegetations. A multimodal imaging approach is increasingly used in the diagnosis of endocarditis and is also part of the diagnostic criteria in the current treatment guidelines, including the use of computed tomography (CT) and nuclear imaging. Exploration into a multimodal approach, particularly, regarding the measurement of vegetation size, is limited. A study identified a substantial correlation in estimating vegetation size using both cardiac CT and TOE (*r* = 0.86; *P* < 0.000001).^18^ However, it is noteworthy that this study exclusively enrolled patients necessitating surgical intervention for IE, potentially excluding individuals with smaller vegetations.

Another study assessed interrater agreement in 30 patients with suspected IE.^19^ TTEs were evaluated by four experienced echocardiographers, who characterized the vegetations by site, size, mobility, shape, embolic risk, and attachment characteristics. Their findings ranged from 98% agreement of the presence of the vegetation to 57% agreement regarding vegetation mobility. Their interrater variability according to the size of the vegetation was fair to good, with complete agreement in 73%. A direct comparison with our findings is difficult. Our study used a numeric scale reporting vegetation size, whereas the other studies published used categorical scales. In our study, the mean vegetation length measured was 15.4 mm. In supplementary analyses, where the original data were categorized in groups of <10, 10–15, and >15 mm, the experts had a complete agreement of 80%, the cardiologists had a complete agreement of 76%, and the novices had a complete agreement of 67%. Some raters might have changed their answers if a categorical scale was used and hence cannot be used as a direct comparison. However, a categorical scale would be especially interesting if the size of the vegetations chosen were centred around 10 mm since guidelines in some cases recommend surgery with vegetations >10 mm.^11,12^

When we compared the three groups with the IE team measurement, our findings suggested that all three groups measured the vegetations smaller than the team. The cardiologists and the experts had a mean underestimation of −2.1 and −2.2 mm, respectively, while the novices had a mean underestimation almost twice as high. Thus, an underestimation might not be uncommon when unexperienced sonographers or doctors are measuring the size of vegetations in TOEs potentially delaying or neglecting necessary surgical intervention. A study from 2016 investigated interrater variability between six site readers and one very experienced echocardiographer from a core laboratory (ECL) on a random sample of 110 echocardiograms.^20^ The inter-site-ECL variability was moderate to excellent for categorical variables regarding vegetations. Vegetation size agreement was fair to good, but no systematic under- or overestimation were reported. Of note, all the site readers in their study were defined as echocardiographic experts which might not reflect clinical daily practice.

Similar to other echocardiographic measurements, one might assume that the precision of individual assessments of vegetation measurements can be enhanced through training. A study conducted by Negishi *et al.*^21^ explored the impact of experience and training on the accuracy of strain measurements, both before and after individualized feedback was provided. Despite the absence of a significant improvement in ICC post-feedback, noteworthy enhancement in segmental strain analysis was observed irrespective of experience. Correspondingly, analogous to the present investigation, their findings indicated significantly lower ICC agreement among less experienced individuals. To date, there exists no inquiry into the effects of training or experience on vegetation measurements in IE patients. The present study did not investigate whether interrater agreement improved throughout the study from the initial to the final vegetation. This decision was motivated by the substantial divergence in image quality within this study. Consequently, any observed differences would likely be attributable to variations in quality rather than indicative of genuine improvement.

One major recommendation in the guidelines is the establishment of a multi-disciplinary endocarditis team where complicated IE patients should be early referred, and noncomplicated cases should be discussed on a regular basis by the team.^9,12^ This is thought to minimize misdiagnosis and the risk of over- or underestimating vegetation size and to determine whether surgery combined with antibiotics or antibiotics alone is the best course of treatment. In our study, we only investigated the interrater variability in one single metric—the vegetation size. The decision whether to operate or not is multifactorial and the vegetation size is rarely the only factor when deciding on the optimal treatment strategy. However, it is undoubtedly a factor prone to error if not managed correctly. A recent study investigated the interrater agreement between two very experienced echocardiographers when measuring vegetation size alone in an offline setting, meaning that the raters were measuring on the same frames.^22^ The disagreement between the operators was on average 3.3 ± 2.9 mm, resulting in a moderate to good correlation (ICC 0.76). Furthermore, the study illustrated that the interobserver variability was high enough to affect surgical intervention in 18 and 25% if vegetation size was the only parameter based on the recommendations of >10 and >15 mm. Similar to our study, they showed a high dispersion within the two raters with a 95% LOA ranging from −9.6 to 7.9 mm. The study was conducted as an offline study which does not reflect daily practice where operators may choose different frames to measure. Our results suggest that the MDC, which denotes an actual change in measurement not due to measurement errors, is 2.6 mm for experts, 3.3 mm for cardiologists, and 3.6 mm for novices. These numbers might be high due to some outliers in every group but could be used to create an interval of possible measurement errors around the 10 mm cut-off, which would be considered a grey area and, therefore, mandatory to consult with a special team.

These findings indicate the difficulties in the assessment and work-up in patients with IE. Hence, we believe that our results underline the need for a multi-disciplinary approach with experts where all patients with IE should be discussed.

### Limitations

There are several limitations to consider. Our selection of echocardiographic exams consisted of patients with definite IE, most of them with large vegetations. Thus, the variance will be larger. In this study, we used a conveniently chosen sampling method. This means, that we intentionally picked a range of echocardiographic images showing different sizes, shapes, and locations of vegetations, and thereby introducing a potential bias into the analysis. We decided to limit the number of TEEs to 20 comprehensive analyses. This decision was driven by our intention to accommodate larger groups of observers, as our main focus in this article was on the differences between groups.

We set a time limit of 3 min per patient which may not reflect a daily clinical practice.

We observed that almost all the experts and cardiologists did not use the full 3 min whereas the novices did. A time limit in this case would have a greater impact on the non-experienced doctors compared with the experienced. However, young unexperienced doctors rarely handle the diagnostic process of IE cases in Denmark, and the inclusion of novices may not have any clinical relevance.

We scheduled and conducted all the measurements in the echocardiographic department at different times of day whenever the rater was available. Few of them performed the measurements during work hours while answering questions from colleagues and taking phone calls, while others did the measurements in a quieter environment after work. The first might reflect a daily working environment as opposed to a standardized protocol. One of the vegetations (number 18) had both vegetation on the mitral valve and an artefact on the tricuspid valve. All the novices measured the actual vegetation while five out of nine experts and five out of nine cardiologists measured the artefact. Lastly, an expectancy bias in this study will be that some of the observers expected that the exams chosen were a part of the ASTERIx project (NCT:05061355) which investigates vegetations between 10 and 30 mm.

## Conclusions

This study showed that measuring vegetation size is an operator and analyser-dependent measurement and should be done with caution. Our study underlines the importance of echocardiographic expertise when evaluating and measuring vegetations and supports the current recommendations that complicated cases, including cases within the 10 mm limit, should be consulted with or cared for by a multi-disciplinary IE team.

## Supplementary Material

qyae024_Supplementary_Data

## Data Availability

The data underlying this article cannot be shared publicly due to the privacy of individuals who participated in the study. The data will be shared on reasonable request to the corresponding author.

## Lead Author Biography

I am Louise Schmidt, a recent graduate of the University of Copenhagen, Rigshospitalet, having obtained my MD degree in 2023. My main interests are centred around echocardiography and its diagnostic role in medicine, particularly, its application in clinical research and in daily patient care settings. This study is the result of a pre-graduate research year at the Department of Cardiology where I worked intensively with the practical use and interpretation of echocardiography in the setting of IE.

I currently work as a medical doctor in an emergency department, but I will be pursuing a residency in cardiology this summer.
