# Supplementary figures and images for: Importance of experience in transoesophageal echocardiographic evaluation of vegetation size in patients with infective endocarditis: a reliability study
Source: Eur Heart J Imaging Methods Pract. 2024 Apr 9;2(1):qyae024. doi: 10.1093/ehjimp/qyae024 (PMC11195693; doi:10.1093/ehjimp/qyae024)

**
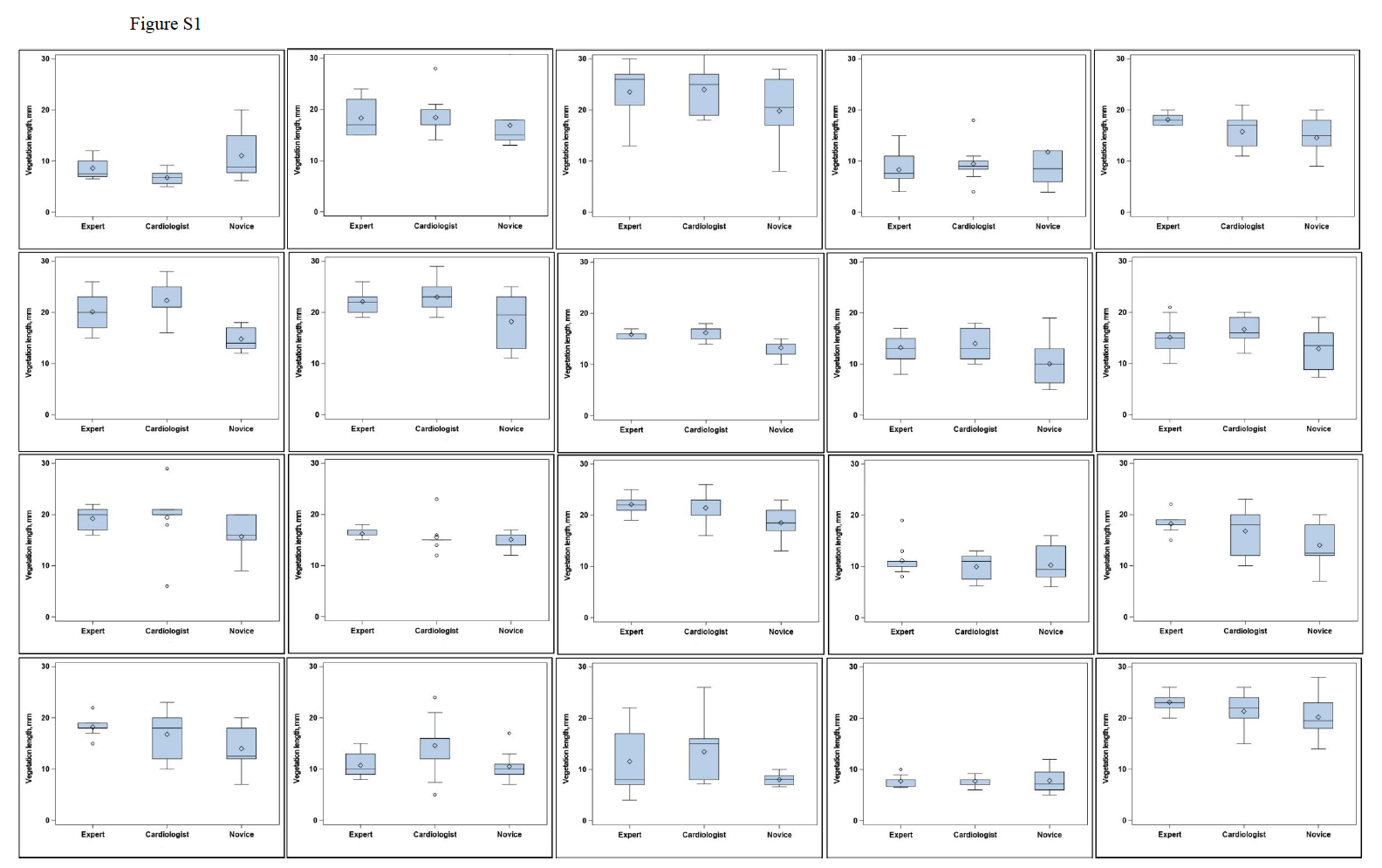
Figure S1**

Supplement: qyae024_Supplementary_Data [file qyae024_Supplementary_Data.docx]
